# Sulfuric Acid for Opening Root-Canals

**Published:** 1895-04

**Authors:** J. R. Callahan


ARTICLE VII.

### SULFURIC ACID FOR OPENING ROOT-CANALS

To make the purpose of this paper clear, let us suppose we have an inferior molar in which the pulp has been destroyed; we adjust the rubber dam, open the pulp-chamber thoroughly, take an old discarded broach, twist a little cotton on the end, bend the broach to a right angle, so that it will reach well down into the cavity; place the broach in a suitable handle, and by means of broach and cotton place directly upon and about the dead pulp a drop or two of a forty or fifty per cent aqueous solution of sulfuric acid. The solution, by a process of dehydration, will cause the pulp to shrink and toughen, so that it can with comparative ease be removed. Now by means of broach and cotton place a drop of the solution over the entrance of each canal; sometimes it will be necessary to sink a little well or depression at the mouth of the canal to get the acid to stay where it is wanted, being careful to use only round or bud drills for this purpose. Take a No 5 Donaldson nerve-canal cleanser, bend it to a suitable angle, cut the shank short with nippers, so that the broach will fit up close to the handle and be rigid and strong; then with a pumping motion begin to enter the canal slowly and carefully. The acid will precede and follow closely the fine broach, and destroy all septic matter with which it comes in contact. Proceed till the patient notifies you of a sensation which is similar to that felt when chloro-percha goes through the foramen. Treat all of the canals in the same manner; I say all, because sometimes you will find what appear to be four distinct canals. Usually three canals will be found; the posterior root will have one broad canal, the anterior root will nearly always show what seem to be two canals. By this time the solution will be so charged with disintegrated tooth- and pulp-substance that it will hide the canals from view. Now by means of a Dunn syringe fill the cavity with a saturated solution of bicarbonate of soda; this when brought in contact with the acid solution liberates carbonic acid gas in such quantities that the effervescence will carry all the broken-up tooth and pulp-substance out of the canal, out of the tooth onto the rubber-dam, leaving a deposite of bicarbonate of soda, lining the whole tooth. This deposit can be removed, if so desired, by a little sterilized water, alcohol, or peroxid, either of which will leave the canals white and clean. If necessary to make the canals larger, place more acid in them and use a larger broach, till the canal is as large as wanted; then cleanse again with bicarbonate of soda, dry the canals thoroughly by means of paper points, alcohol, hot air, etc., and you have the cavity and all the canals thoroughly opened, thoroughly clean, thoroughly aseptic, so that you can proceed to treat or fill as you choose. I usually fill the roots at once, but if there be signs of inflammation, I treat according to symptoms.

There are numerous cases where experience has shown this treatment to be of great value. I will relate briefly the case of Miss A., who had called on her regular dentist for relief; she was annoyed by a soreness about the roots of the right inferior second molar. The dentist, a very skillful man, tried in every way he knew to give her relief, but with all his skill and perseverance he failed to get the apical foramen open, so that the pus might escape from the apical space. Cutting through the gum and bone to the apex of the root was suggested, but the tooth had grown so sore by this time that the lady lost courage and refused to submit to the operation. Upon the advice of a neighbor, she called at my office. By the use of the acid and broach the pus was escaping through the root canals in a very few minutes, and the roots were filled at the second sitting. Some of you no doubt are ready to declare such treatment will ruin the tooth and damage the surrounding tissues; to which I answer, nearly five years' use in my own hands and one to three years' use in the hands of several of my professional friends has produced results far more satisfactory to ourselves and our patients than we are able to obtain by the usual methods.

Allow me to give in a condensed manner a few reasons for my faith in this seemingly heroic treatment.

First, the action of sulfuric acid on dentine is self-limiting, at least so nearly so as to make the operation perfectly safe.

Second, $H_2SO_4$ is a pronounced germicide.

Third, it has been shown that sulfuric acid acts with far greater vigor upon diseased tissue than upon healthy tissue.

Fourth, the acid breaks down or destroys the diseased tissue, leaving a fresh aseptic surface that nature will take care of with but little assistance; this condition obtains in the abscess and fistulous tract, as well as in the root.

Fifth, an aseptic wound will heal itself in any part of the body, if properly closed.

Sixth, the solution softens the dentine for an indefinitely short distance; the broach removes this softened denting, the acid instantly attacks the fresh surface, the instrument again cutting the softened surface,and so on, as long as the tooth is exposed to the action of the instrument and the acid.

Seventh, the bicarbonate-of-soda solution neutralizes the acid, and at the same time generates carbonic acid gas in sufficient quantities to carry the *debris* from the canals.

I have tried to avoid making Munchausen-like statements as to the infallibility of this method in each and every inaccessible and spiral-like root canal, but I will promise those who carefully and intelligently undertake this work an agreeable surprise. You will be surprised at the ease, the brevity, the thoroughness, and the permanent success attending the intelligent use of this method. —Callahan (J. R.), *Cosmos.*

